# Acupuncture for Poststroke Cognitive Impairment Based on Default Mode Network Analysis: Protocol for a Randomized Controlled Trial

**DOI:** 10.2196/74981

**Published:** 2025-09-12

**Authors:** Wangxinjun Cheng, Moyu Wang, Moyi Li

**Affiliations:** 1 Department of Rehabilitation Medicine First Affiliated Hospital Jiangxi Medical College, Nanchang University Nanchang China; 2 Queen Mary College Nanchang University Nanchang China

**Keywords:** cognitive function, traditional Chinese medicine, functional magnetic resonance imaging, fMRI, randomized controlled trial, poststroke cognitive impairment, PSCI

## Abstract

**Background:**

Poststroke cognitive impairment (PSCI) is a prevalent and disabling complication following stroke, affecting critical functions such as memory, attention, language, and executive abilities. Despite the growing clinical burden, standardized and effective treatment strategies for PSCI remain limited. Acupuncture, a key modality in traditional Chinese medicine, has shown promise in improving cognitive outcomes among survivors of stroke. However, the neural mechanisms underlying its efficacy are not well understood. The default mode network (DMN), a brain network implicated in cognition and memory, has been shown to exhibit altered functional and structural connectivity in patients with PSCI. Investigating whether acupuncture modulates DMN activity may provide critical insights into its therapeutic potential.

**Objective:**

This study aims to evaluate the efficacy of acupuncture in improving cognitive function in patients with PSCI and explore its potential neurobiological mechanisms, particularly those involving changes in the DMN, using multimodal neuroimaging techniques.

**Methods:**

We will conduct a single-blind, randomized controlled trial involving 54 eligible patients with PSCI who will be randomly assigned to either an acupuncture group or a sham acupuncture control group. Both groups will receive conventional rehabilitation therapies. The intervention group will undergo standardized scalp acupuncture targeting Baihui (GV20), Shenting (GV24), and Sishencong (EX-HN1) for 8 weeks. The control group will receive sham acupuncture at nonacupoint locations using placebo needles. Cognitive function will be assessed at baseline and 4 and 8 weeks using the Montreal Cognitive Assessment and Mini-Mental State Examination. Secondary outcomes include activities of daily living, quality of life, and neuroimaging data acquired through resting-state functional magnetic resonance imaging and diffusion tensor imaging.

**Results:**

This study is currently in the recruitment phase. All results, including clinical and imaging data, will be reported upon trial completion and publication.

**Conclusions:**

This protocol is designed to investigate the efficacy of acupuncture and its underlying mechanisms in treating PSCI, with a particular focus on functional brain networks. By integrating clinical cognitive assessments and neuroimaging analysis of DMN connectivity, this study seeks to establish objective correlates of cognitive improvement. Findings from this research may advance the understanding of how acupuncture modulates large-scale brain networks and contribute to the development of imaging-based biomarkers for treatment evaluation. If successful, this approach could support the inclusion of acupuncture as a personalized nonpharmacological strategy in the neurorehabilitation of cognitive deficits following stroke.

**International Registered Report Identifier (IRRID):**

PRR1-10.2196/74981

## Introduction

### Background

Stroke stands as a prominent global contributor to disability and mortality. Annually, it afflicts 13.7 million individuals and claims 5.5 million lives [1]. Often accompanied by limb hemiplegia, aphasia, dysphagia, and other neurological dysfunctions, stroke is responsible for a high likelihood of cognitive impairment [2]. In 2016 alone, the world witnessed 13.7 million new incident strokes [3], with an annual incidence ranging from 150 to 200 per 100,000 people [4]. Poststroke cognitive impairment (PSCI) refers to a range of cognitive deficits that may develop in patients following a stroke. It can manifest as poststroke dementia, which occurs in up to a third of patients within a year after the stroke and is closely linked with increasing age [5]. The underlying pathology of PSCI is heterogeneous, with many cases stemming from various vascular etiologies and brain changes in addition to degenerative processes. PSCI encompasses conditions such as multi-infarct dementia, strategic infarct-related dementia, subcortical ischemic vascular dementia, and Alzheimer disease [6]. It emerges as a common complication, with studies reporting the combined prevalence of PSCI as 39% to 47% [7]. Three months after stroke, dementia develops in 6% to 32% of patients, elevating the risk of dementia in survivors of stroke to 3.5 to 5.6 times that of the general population. The associated cognitive dysfunction significantly diminishes quality of life for affected individuals [8]. Evidence-based medical research underscores that cognitive dysfunction exerts a more enduring impact on survivors of stroke than physical dysfunction [9], making cognitive rehabilitation a pressing research focus.

Presently, there is a lack of a standardized and effective treatment plan for PSCI, and exploration into rehabilitation methods for cognitive function continues [10]. While cognitive training is widely used and has shown positive therapeutic outcomes, its effectiveness relies on the patient’s cognitive condition, and it typically does not maintain long-term benefits [11,12]. Emerging technologies such as cranial magnetic stimulation and virtual reality hold promise, yet they remain unstandardized, their costs remain high, and widespread implementation remains challenging [13]. In contrast, traditional Chinese medicine (TCM) therapy, notably acupuncture, has gained international recognition. Numerous studies attest to acupuncture’s efficacy in treating cognitive dysfunction [14-16].

Moreover, investigations into the regulatory mechanisms of acupuncture on cognitive function have yielded insightful findings. For instance, Lu et al [17] observed that acupuncture enhances blood perfusion and glycol metabolism in specific brain regions, positively impacting cognition. Huang et al [18], using a combination of positron emission tomography and computed tomography, demonstrated that acupuncture exerts a regulatory effect on brain functional areas, potentially contributing to poststroke patient rehabilitation.

According to the principles of TCM, the brain marrow serves as the material foundation for cognitive maintenance. Kidney essence deficiency can lead to insufficient brain marrow, potentially causing cognitive decline. It may be attributed to mild cognitive impairment (MCI) induced by renovascular hypertension [19]. On the basis of previous research findings, Baihui (GV20), Shenting (GV24), and Sishencong (EX-HN1) are closely associated with improvements in brain and cognitive functions. These acupoints are commonly selected in basic research on ischemic stroke, autism, depression, and other related conditions [20,21]. The acupoint Baihui (GV20) is believed to connect the governor vessel with the brain and is closely associated with brain function. Baihui (GV20) and Touqu (GB7) acupuncture alleviates neurological deficits by reducing oxidative stress and neuronal apoptosis via the SIRT1/FOXO1 pathway [21]. Shenting (GV24), also part of the governor vessel, is described in Huang-Fu Mi’s *The* *Systematic Classic of Acupuncture and Moxibustion* as the intersecting point of the bladder meridian of foot taiyang, the stomach meridian of foot yangming, and the Du meridian, with effects that include calming the mind and stimulating the brain, making it useful for treating mental and cognitive disorders. Sishencong (EX-HN1), an extra point located on the neck and head, is described in the *Taiping Shenghui Fang* as having the ability to tranquilize and enlighten the mind. Together, Baihui, Shenting, and Sishencong are thought to enlighten and awaken the mind of patients experiencing cognitive impairment. In recent years, acupuncture—a key modality of TCM—has gained increasing global recognition for its potential in cognitive rehabilitation. Clinical studies have reported that acupuncture may improve cognitive function in individuals with MCI, Alzheimer disease, and PSCI [16,22,23].

Neuroimaging and metabolic studies have suggested that acupuncture may exert regulatory effects on brain regions associated with cognition. For instance, Ma et al [7] conducted a meta-analysis of functional magnetic resonance imaging (fMRI) studies and found that acupuncture modulates activity in the prefrontal cortex, hippocampus, thalamus, and default mode network (DMN) in patients with MCI. Furthermore, a systematic review by Li et al [24] confirmed that resting-state fMRI studies consistently support acupuncture’s regulatory effects on intrinsic brain connectivity patterns related to cognition, attention, and memory. On the basis of both clinical efficacy and neuroimaging evidence, this study selected Baihui (GV20), Shenting (GV24), and Sishencong (EX-HN1) as the primary intervention points. These acupoints, located on the scalp overlying key cognitive and prefrontal regions, have been frequently used in previous studies on cognitive dysfunction. While TCM theory historically associates these points with mental clarity and brain function, their selection in this study is further supported by emerging neurobiological findings indicating potential roles in modulating the central nervous system.

In recent years, neuroimaging technologies have played an increasingly vital role in advancing our understanding of brain diseases. Among them, fMRI has emerged as a particularly valuable tool due to its ability to noninvasively measure brain activity and connectivity [25]. In the context of PSCI, fMRI provides high spatial resolution and can detect subtle changes in brain networks associated with cognitive processes [26,27]. Specifically, resting-state fMRI allows for the assessment of intrinsic functional connectivity without the need for active task performance—an important consideration given the cognitive and physical limitations of patients with PSCI.

The DMN, a brain network closely linked to cognition and memory, is often disrupted in individuals with PSCI [28]. fMRI enables the direct observation of DMN connectivity patterns, offering a unique opportunity to investigate how interventions such as acupuncture might modulate these networks [29]. Previous studies have demonstrated that acupuncture can influence brain activity and connectivity, including within the DMN, suggesting a potential mechanism through which acupuncture may improve cognitive function. Ellwood-Lowe et al [30] indicate that the lateral frontoparietal network and the DMN are associated with cognitive test performance. The findings of Liu et al [31] demonstrate that the functional connectivity of DMN in patients with cognitive dysfunction after stroke differs from that of healthy individuals and correlates with variations in memory function. In addition, reduced feeder and local connections have been observed within cognition-related networks, including the DMN, the salience network, the cingulate network, and the orbital network, particularly involving the caudate nucleus [32]. Notably, changes in executive function and resting-state functional connectivity are common after stroke or ischemic attacks, indicating a connection between cerebrovascular events and the prolonged impairment of neural network connectivity and cognitive function [33]. Therefore, our hypothesis posits that acupuncture might ameliorate the cognitive function of patients with PSCI by modulating the DMN. However, no studies have investigated the mechanism of acupuncture in enhancing the cognitive function of patients with PSCI from the perspective of DMN brain structure and function.

As a consequence, we are adopting a randomized controlled trial methodology for this investigation. Participants with diagnosed PSCI will be randomly assigned to either an acupuncture intervention group or a sham acupuncture group. The efficacy of acupuncture as a therapeutic modality will be evaluated through the use of standardized cognitive function scales, which will provide quantitative evidence of its clinical rehabilitative potential in enhancing cognitive function in patients with PSCI. In parallel, we will use fMRI to elucidate the neuroimaging mechanisms underlying the therapeutic effects. The objective of this research is to establish a standardized cognitive rehabilitation protocol informed by these neuroimaging insights for the treatment of PSCI.

### Objectives

The primary objective is to test the hypothesis that acupuncture, compared with conventional treatment, will lead to significant improvements in poststroke cognitive function as measured using standardized neuropsychological assessments. A secondary hypothesis is that acupuncture will modulate the functional connectivity within the DMN as detected by resting-state fMRI and that changes in DMN connectivity will correlate with improvements in cognitive performance.

## Methods

### Study Design

A randomized, single-blind, parallel-controlled trial will be undertaken to assess the impact of acupuncture on enhancing cognitive function in individuals with PSCI. A total of 54 participants will be recruited and randomly allocated to either the acupuncture group or the conventional treatment control group. The intervention will span 8 weeks, with cognitive evaluations scheduled at baseline, 4 weeks after treatment, and 8 weeks after treatment. Previous trials have shown that such frequency and intensity of treatment are both safe and effective in improving cognitive outcomes in survivors of stroke [34,35]. Participant attendance will be closely monitored. Any missed sessions will be documented along with reasons, and efforts will be made for rescheduling. Treatment logs will be maintained by treating physicians to record each session, including date, time, and whether the session was completed as scheduled.

### Sample Size

The primary outcome of this study is the change in cognitive function as measured using the Beijing version of the Montreal Cognitive Assessment (MoCA). We prespecified a standardized between‑group effect size of *d*=0.40 on the MoCA at 8 weeks. This assumption is supported by a recent meta‑analysis of acupuncture for PSCI showing a pooled mean difference (MD) of 3.30 points (95% CI 2.19-4.40) versus control [36,37]. Using representative poststroke variability (MoCA score mean 21.1, SD 7.5), an MD of 3.30 corresponds to a standardized effect of approximately 0.44; thus *d*=0.40 is conservative. Therefore, the trial is powered on MoCA as the primary cognitive end point.

Using G Power (version 3.2.7) with a 2-tailed test, α set at.05 (type I error), and power (1 – β) at 0.80 (type II error β=.20), the minimum required sample size was calculated to be 44 participants (22 per group) [38]. To account for an anticipated 20% dropout or loss to follow-up rate, the total sample size was increased to 54 participants, with 27 participants allocated to each group.

### Participants and Recruitment

**Figure 1:**
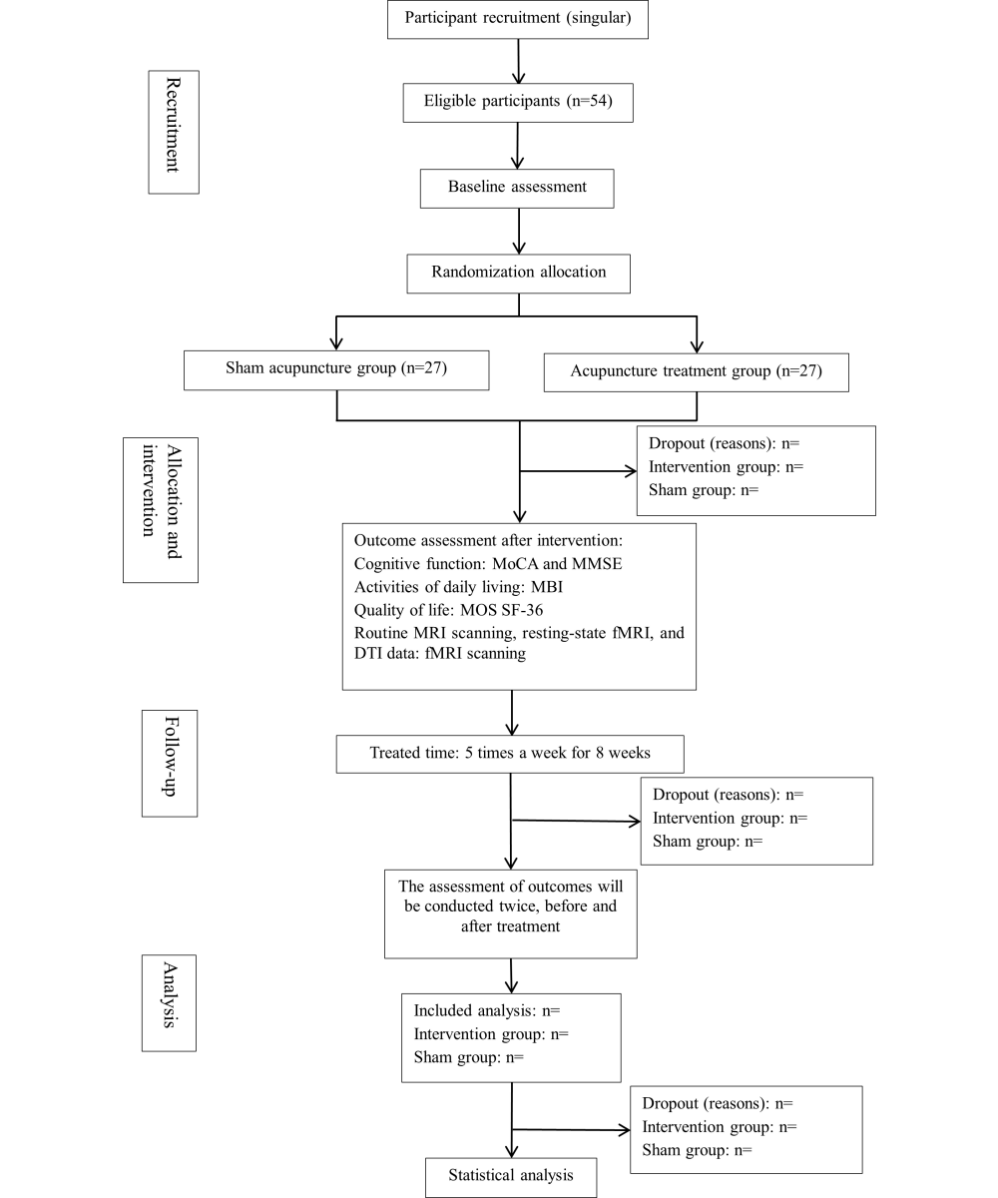
Flow diagram of participants. DTI: diffusion tensor imaging; fMRI: functional magnetic resonance imaging; MBI: modified Barthel index; MMSE: Mini-Mental State Examination; MoCA: Montreal Cognitive Assessment; MOS SF-36: Medical Outcomes Study 36-Item Short-Form Health Survey; MRI: magnetic resonance imaging.

We aim to enroll 54 participants through a combination of ward visits and online promotional efforts. Individuals expressing interest in participating will undergo eligibility assessment conducted through a screener, typically via contact at the recruitment office. A MoCA score of <26 is used as the primary cutoff to identify cognitive impairment in accordance with the standard diagnostic threshold recommended in clinical guidelines and previous studies. Patients scoring below this threshold are considered eligible for inclusion. In cases of low educational level (≤12 years), 1 point is added to the total MoCA score as per standard adjustment procedures. Should the applicant satisfy the research criteria, an invitation to partake in the study will be extended. Figure 1 shows the flow diagram of participants.

### Eligibility Criteria

Eligible participants must meet the specified inclusion criteria and not fall under the exclusion criteria. The inclusion criteria are as follows: confirmation of the diagnosis through adherence to the stipulated criteria and validation via head magnetic resonance imaging (MRI) or computed tomography examination; disease duration of >1 month; first ever stroke to avoid confounding effects of previous cerebrovascular events on brain network reorganization and cognitive function; conscious state with stable vital signs and nonprogressive symptoms; clear consciousness without meeting the criteria for *dementia* (Mini-Mental State Examination [MMSE] score of >17 for illiterate individuals, >20 for individuals with a primary school educational level, and >24 for individuals with a middle school educational level or higher—thresholds selected to ensure that participants can complete the full range of cognitive assessments while still presenting mild to moderate PSCI); onset of cognitive impairment after stroke; age of >18 years; right-handedness to reduce variability in brain lateralization patterns, which is particularly important for imaging analyses; and voluntary participation with the provision of self- or legal guardian–signed informed consent.

Exclusion criteria encompass recurrent disease; cognitive impairment originating from other causes; the presence of severe conditions such as liver, kidney, or heart disease; and impediments to cooperation with examinations due to severe hearing impairment, language barriers, or other significant reasons. In addition, all potential participants will be assessed using validated screening tools such as the Hospital Anxiety and Depression Scale or Hamilton Depression Rating Scale at baseline. Patients who meet the diagnostic threshold for moderate to severe depression or anxiety (Hospital Anxiety and Depression Scale score of ≥11 or Hamilton Depression Rating Scale score of ≥17) will be excluded from the trial to avoid emotional state confounding cognitive outcome assessments.

### Randomization and Allocation Concealment

An impartial statistician will generate a concealed random allocation sequence using the PLAN procedure in the SAS statistical software (version 9.1; SAS Institute). Eligible participants will be randomly assigned in a 1:1 ratio to either the acupuncture treatment group or the conventional treatment control group. The random sequence will be securely stored and managed by a project manager who is not involved in participant recruitment, screening, or outcome assessment. To ensure allocation concealment, the group assignments will not be disclosed to screeners, evaluators, or participants during the enrollment process. Once a participant is deemed eligible and enrolled, the project manager will inform them of their group assignment via telephone, thus maintaining strict separation between the randomization process and other trial operations.

Given the nature of acupuncture, it is not feasible to blind the practitioners delivering the intervention. We acknowledge that the lack of practitioner blinding could introduce performance bias and influence internal validity through potential nonspecific or placebo effects. To minimize these risks, we will (1) blind participants to the specific study hypotheses and expected effects of each intervention, (2) ensure that outcome assessors and statisticians remain blinded throughout the trial, (3) standardize the practitioner-patient interaction by using scripted instructions and limiting nonprotocol communication, and (4) provide the control group with conventional treatment to control for attention and treatment expectancy effects. These measures aim to reduce expectancy bias and placebo responses, thereby strengthening the internal validity of the trial despite the inherent limitations of acupuncture blinding.

### Blinding

This study will use a single-blind design in which both the participants and the primary outcome assessors will remain blinded to group assignments. A designated project manager will oversee the generation of the randomization sequence and the allocation of blinding codes. Group assignments will be generated using a computer-based random number table and anonymized as group A or B.

Given the inherently distinguishable nature of acupuncture interventions, blinding of treating physicians is not feasible. However, all treating physicians will undergo standardized training to ensure consistent procedures and neutral communication, thereby minimizing observer bias.

Participants will be randomly assigned to either the acupuncture group or the conventional treatment group. To maintain participant-level blinding, the conventional treatment group will receive sham acupuncture using specially designed nonpenetrating needles applied to nonacupoint locations. The procedures, including manipulation, duration, and frequency, will be identical in both groups to ensure comparable treatment experiences.

Outcome assessors and statistical analysts will be blinded to the actual group identities and will only have access to anonymized labels (A or B). Conversely, treating physicians and project managers will not participate in outcome assessment or data analysis. Similarly, outcome assessors and analysts will not be involved in participant enrollment or allocation.

The blinding code will be securely sealed and will not be disclosed until the completion of all statistical analyses.

To assess the success of blinding, participants will be asked at the end of the treatment period to indicate which group they believe they were assigned to (A, B, or unsure) via a structured questionnaire.

### Intervention

All participants will receive conventional therapy standardized according to the Chinese guidelines for the diagnosis and treatment of vascular cognitive impairment (2024 edition) [39], with clearly defined components, treatment frequency, and documentation procedures to ensure reproducibility.

#### Conventional Treatment Group

##### Basic Medical Treatment

Standard pharmacological management will follow the 2024 Chinese guidelines, including risk factor control. Medication type and dose will be individualized based on comorbidities and physician judgment, but treatment categories, target parameters (blood pressure of <140/90 mm Hg and low-density lipoprotein cholesterol level of <2.6 mmol/L), and monitoring schedules will be uniform across participants.

##### Basic Rehabilitation Therapy

A standardized rehabilitation framework will be applied, consisting of three core modules:

1. Exercise therapy: 30 minutes per session, 5 sessions per week, including balance training, gait training, and limb mobility exercises. Intensity will be tailored to baseline motor ability using the Fugl-Meyer Assessment, but progression will follow a predefined stepwise protocol.
2. Occupational therapy: 30 minutes per session, 5 sessions per week focusing on activities of daily living such as dressing, feeding, and household tasks using standardized task sequences.
3. Physical agent modality therapy: where indicated, treatments such as transcranial direct current stimulation or low-frequency pulsed magnetic therapy will be applied following fixed device settings and session lengths.

##### Cognitive Function Training

Structured cognitive training will target attention, memory, judgment, reasoning, and executive function. Each session will last 30 minutes once daily, 5 days per week for 8 weeks, delivered using a standardized digital training program and exercise booklet. Therapists will follow a fixed progression schedule, with difficulty levels increasing every 2 weeks.

##### Sham Acupuncture Procedure

Sham acupuncture will be administered at nonacupoint sites approximately 2 cm posterior to Baihui (GV20), 1.5 cm lateral to Shenting (GV24), and 2 cm adjacent to Sishencong (EX-HN1), avoiding recognized meridians [40]. A nonpenetrating placebo needle will provide superficial tactile pressure without skin penetration, mimicking real acupuncture sensation but avoiding physiological effects [41]. Sessions will last 20 minutes once daily, 5 days per week for 8 weeks.

#### Acupuncture Treatment Group

##### Overview

Participants will receive all the aforementioned conventional therapy components and real acupuncture in place of sham acupuncture. Selected acupoints will include GV20, GV24, and EX-HN1. Disposable sterile filiform needles will be inserted to a depth of 10 to 15 mm and manipulated to elicit *deqi* sensation. Each session will last 20 minutes once daily, 5 days per week for 8 weeks.

##### Standardization and Quality Control

All therapists and acupuncturists will undergo a 2-day training workshop on the standardized protocol. Detailed treatment manuals and checklists will be used to guide therapy delivery. Treatment logs will be completed after every session, documenting date, time, duration, completion status, and any deviations from the protocol. Monthly audits will be conducted by the project manager to ensure adherence to the protocol.

### Primary Outcomes

All practitioners involved in the study are experienced and professionally trained rehabilitation physicians or therapists. The MoCA will serve as the primary outcome measure for statistical inference as it is more sensitive to detecting MCI and changes in executive and visuospatial domains commonly observed after stroke. The MMSE will be analyzed as a key secondary outcome to provide complementary information and enable comparison with previous literature where the MMSE was the predominant tool.

Both assessments will be administered by the same trained therapist before and after the intervention to ensure consistency and minimize interrater variability. The MoCA evaluates a broad range of cognitive domains, including visuospatial and executive abilities, naming, memory, attention, language, abstraction, delayed recall, and orientation. It consists of 12 subitems, with a total score of 30 points; a score of >26 is generally considered healthy. For participants with <12 years of education, 1 point will be added to the total score to adjust for education bias.

The MMSE also provides a total score of 30 points and assesses domains such as orientation, registration, attention and calculation, recall, language, and praxis. While the MMSE is widely used in clinical settings for its brevity and utility in detecting moderate to severe cognitive impairment, the MoCA is more sensitive to subtle cognitive changes, making it more suitable as the primary statistical end point in this trial.

Multiple comparisons will be addressed by applying Bonferroni or other appropriate correction methods for secondary outcomes, ensuring statistical rigor while capturing a comprehensive and nuanced picture of cognitive changes following acupuncture intervention. The timing of treatment assessments and data collection are shown in Table 1.

**Table 1:** Timing of treatment assessments and data collection.

Item			Study period				
			Before enrollment (−1 wk)	Baseline (0 wk)	Intervention period		
					4 wk		8 wk
Inclusion criteria			✓				
Exclusion criteria			✓				
Informed consent			✓				
Randomization				✓			
Allocation				✓			
**Interventions**							
	Acupuncture treatment group				✓	✓	
	Sham acupuncture group				✓	✓	
**Assessments**							
	MMSE^a^			✓	✓	✓	
	MoCA^b^			✓	✓	✓	
	MBI^c^			✓	✓	✓	
	MOS SF-36^d^			✓	✓	✓	
	Routine MRI^e^ scanning			✓	✓	✓	
	Resting-state fMRI^f^			✓	✓	✓	
	DTI^g^			✓	✓	✓	

^a^MMSE: Mini-Mental State Examination.

^b^MoCA: Montreal Cognitive Assessment.

^c^MBI: modified Barthel index.

^d^MOS SF-36: Medical Outcomes Study 36-Item Short-Form Health Survey.

^e^MRI: magnetic resonance imaging.

^f^fMRI: functional MRI.

^g^DTI: diffusion tensor imaging.

### Secondary Outcomes

#### Ability to Perform Activities of Daily Living

The modified Barthel index will be used for pre- and posttreatment assessments. Scoring is as follows: 100 points indicate normal ability to manage daily-life activities; scores of >60 imply mild dysfunction and mild life dependence, scores of 41 to 59 indicate moderate dysfunction and moderate life dependence, scores of 21 to 40 reflect severe dysfunction with evident life dependence, and scores of <20 denote total life dependency.

#### Quality of Life

The Medical Outcomes Study 36-Item Short-Form Health Survey will be used for evaluation before and after treatment. This comprehensive scale covers 8 domains, encompassing physical functioning, role limitations due to physical health problems, bodily pain, general health perceptions, vitality, social functioning, role limitations due to emotional problems, and mental health.

#### fMRI Scanning Scheme

A Siemens MAGNETOM Trio 3.0T magnetic resonance imager will be used for routine MRI scanning, resting-state fMRI, and diffusion tensor imaging (DTI) data collection. Both pre- and posttreatment scans will be conducted using this state-of-the-art imaging technology. For imaging data, false discovery rate correction will be used.

Conventional MRI scan sequence and parameters are as follows: (1) repetition time (TR)=600 ms, echo time (TE)=10 ms, layer thickness=5 mm, field of view (FOV)=240 mm, and matrix size=256 × 256 (T1-weighted image parameters); (2) TR=3000 ms, TE=113 ms, layer thickness=5 mm, FOV=240 mm, and matrix size=256 × 256 (T2-weighted image parameters); and (3) TR=8800 ms, TE=79 ms, layer thickness=5 mm, FOV=240 mm, and matrix size=256 × 256 (T2-weighted fluid-attenuated inversion recovery parameters).

The scanning sequence and parameters for fMRI are as follows. First, the anatomical data are collected via a T1-weighted 3D spoiled gradient–recalled sequence, and the volume images are obtained. The scanning parameters are TR=1900 ms, TE=2.26 ms, layer thickness=1 mm, FOV=250 mm, and matrix size=256 × 256. Second, the resting fMRI data are collected via real-time imaging processing. Gradient-recalled echo-planar imaging recalls the image with the following parameters: TR=2000 ms, TE=30 ms, continuous scanning of 30 layers, layer thickness=4 mm, FOV=200 mm, matrix size=64 × 64, and scanning time of approximately 8 minutes and 6 seconds.

The scanning sequence and parameters for DTI are as follows. First, the anatomical data are collected via a T1-weighted 3D spoiled gradient–recalled sequence, and the volume images are obtained. The scanning parameters are TR=1900 ms, TE=2.26 ms, layer thickness=1 mm, FOV=250 mm, and matrix size=256 × 256. Second, the DTI data are scanned through an echo-planar imaging sequence in the horizontal axis. The following parameters**―**TR=7200 ms, TE=104 ms, 49 layers, 2.5-mm thickness, number of excitations once, matrix size=128 × 128, FOV=240 × 240 mm, and 64 nonlinear diffusion gradients (b=0.1000 seconds per mm^2^)**―**are applied. The scanning duration is approximately 8 minutes and 11 seconds.

### Safety Measurements

Acupuncture typically does not lead to severe adverse events [42,43]. However, given the neurological population and the invasive nature of acupuncture, a formal data safety monitoring plan will be implemented. An independent data safety monitoring board (DSMB), comprising a neurologist, an experienced acupuncturist not involved in the trial, and a biostatistician, will oversee participant safety throughout the study. The DSMB will review cumulative safety data and adjudicate all adverse events and serious adverse events independently of the trial investigators, including assessment of causality, severity, and relatedness to the intervention.

All unexpected adverse events will be promptly reported by clinical staff to the investigator or project manager and documented in detail. These reports will be forwarded to the DSMB for independent review within 72 hours. Should a serious adverse event occur, the principal investigator and ethics committee will be notified immediately, and the DSMB will make recommendations on whether the participant should be withdrawn from the trial or whether trial procedures require modification. The DSMB will meet quarterly or more frequently if safety concerns arise to ensure continuous monitoring of participant well-being and trial integrity.

### Data Management

During participant recruitment, screeners will gather demographic and baseline characteristic data. The final assessor will measure primary and secondary outcomes at baseline and at the conclusion of the 8-week intervention. A research assistant will oversee quality control during data collection and take charge of data entry. The project manager will assume responsibility for the initial data cleaning, identification, encoding, and transformation into the suitable format for subsequent data analysis. All study data will be entered into an electronic database with range and consistency checks. Data access will be restricted to authorized study staff. Missing data will be minimized through real-time monitoring and reminders. If outcome data are missing, multiple imputation by chained equations will be applied under the assumption of being missing at random. Pooled estimates across imputations will be reported, with sensitivity analyses using complete-case data.

### General Data and Efficacy Indicator Statistical Analysis

All statistical analyses will be conducted in SPSS (version 20.0; IBM Corp), with the significance level set at *P*<.05. Analyses will be divided into two categories: (1) general data and efficacy indicators and (2) fMRI data processing and statistical analysis. For general data and efficacy indicators, two-tailed independent sample *t* tests or chi-square tests will be used to assess pre- and posttreatment differences in overall cognitive function, activities of daily living, and quality of life between groups.

### fMRI Preprocessing

fMRI data will be preprocessed using the DPABI toolbox a widely used open-source platform for resting-state fMRI analysis that integrates functions such as slice timing correction, head motion realignment, normalization to Montreal Neurological Institute space, spatial smoothing, and nuisance signal regression. DPABI is based on SPM12 (Wellcome Department of Imaging Neuroscience at University College London) and MATLAB (R2023a; MathWorks). Functional connectivity and amplitude of low-frequency fluctuation analyses will be conducted in this study [44].

### fMRI Statistical Analysis

After preprocessing, statistical analyses will be conducted with *P*<.05 as the significance threshold applying false discovery rate correction for multiple comparisons. The analysis will include the following:

1. DMN extraction: random 2-sample *t* tests will compare extracted DMN data between groups, with *P*<.05 considered statistically significant.
2. Functional connectivity of the DMN: the posterior cingulate cortex, medial prefrontal cortex, and medial temporal lobe were defined as regions of interest (ROIs). Resting-state functional connections between ROIs will be calculated, and the time correlation coefficient will be obtained for the 2–independent sample *t* test.
3. Structural connectivity of the DMN: fiber bundles between ROIs will be extracted from DTI data. The average fiber bundle length (mL), average diffusion rate (MD), and mean fractional anisotropy (mFA) will be calculated for each group and compared using 2-sample *t* tests.
4. Correlation analysis: using correlation tests, MoCA, MMSE, MBI, and Medical Outcomes Study 36-Item Short-Form Health Survey scores; *r* values; and mL, mFA, and MD values will undergo statistical analysis. The Pearson correlation coefficient (*r*) will be obtained for the *t* test.

Multiple linear regression models will be used to examine associations between groups (acupuncture vs sham acupuncture) and posttreatment cognitive outcomes (MoCA and MMSE), adjusting for baseline covariates. Logistic regression will analyze dichotomized outcomes, and interaction terms will test moderating effects of demographic and clinical variables.

### Ethical Considerations

This informed consent agreement is executed in adherence to the Declaration of Helsinki. The study protocol and consent form have received approval from the ethics committee of the First Affiliated Hospital of Nanchang University (approval 2020 12-86). All participants will receive comprehensive information about the trial and provide their informed consent through a signed agreement before taking part. All personal information will be kept confidential ― identifiable data will be stored separately from study data, replaced by coded IDs, and accessible only to the research team. Electronic records will be stored on encrypted, password-protected servers and paper forms in locked cabinets. No identifiable information will be reported in publications.

Any significant modifications to the protocol, such as changes to eligibility criteria, outcomes, or analysis plans, will be submitted to the research ethics committee for review and approval before implementation. The approved amendments will also be communicated promptly to all relevant parties, including the trial investigators, trial participants, trial registries, and relevant journals or regulatory bodies. These modifications will also be documented in an amendment log and will be audited at least annually to ensure consistency across the registered protocol and study documents.

### Trial Status

This protocol is version 1, dated August 20, 2025. The trial has not yet started.

## Results

We are currently in the recruitment phase, and we will publish the results in open-source journals when the trial is completed. As of August 2025, we have completed protocol finalization and ethics approval. Recruitment has begun, and no participants have been enrolled yet.

## Discussion

### Therapeutic Approaches

Currently, cognitive training stands as the predominant rehabilitation therapy for PSCI, demonstrating positive effects across various cognitive domains. Nevertheless, its efficacy is constrained by high demands on patients’ cognitive function, limiting its application in cases of severe cognitive dysfunction. Alternative therapies, including TCM, exercise, and music therapy, exhibit some potential in improving the cognitive function of patients with PSCI, yet their effectiveness remains unclear. Acupuncture, rooted in TCM principles, has been recognized by scholars for its positive impact on alleviating cognitive dysfunction in patients with PSCI. Notably, studies suggest that specific acupuncture points such as Baihui, Shenting, and Sishencong significantly enhance cognitive function in patients with PSCI [45,46]. However, the precise mechanisms through which acupuncture modulates brain function in patients with PSCI, particularly in the DMN brain region associated with cognitive function, remain unexplored.

In this trial, we have implemented a stringent randomized controlled study design, wherein patients diagnosed with PSCI are randomly allocated to either a sham acupuncture group or an acupuncture treatment group. Although cognitive function assessments typically involve subjective scales, we are enhancing the consistency and reliability of these assessments by providing standardized training for the evaluators and ensuring that each patient is assessed by the same evaluator. Furthermore, fMRI will be used to explore the neuroimaging mechanisms that underpin the effects of acupuncture, with the goal of offering valuable insights for clinical practice.

**Brand New Insights**

A suite of analyses, including independent component analysis, functional connectivity analysis, and DTI analysis, will be conducted before and after the intervention to assess changes in activation patterns, functional connectivity, and structural connectivity within brain regions associated with the DMN. Correlation analyses between changes in DMN and cognitive function will be conducted to elucidate the neuroimaging correlates of the structural and functional changes within the DMN.

Crucially, all participants will receive standardized cognitive rehabilitation as part of routine clinical treatment throughout the trial, with both the acupuncture treatment group and the sham acupuncture group undergoing the same treatment regimen. This design intentionally sets cognitive training as a constant therapeutic background to isolate the specific effects of acupuncture from nonspecific placebo responses. Therefore, the observed differences between groups will solely represent the contribution of acupuncture points to neuropsychology rather than absolute treatment outcomes. This method rigorously controls cognitive training variables, precisely quantifies the incremental benefits of acupuncture based on evidence-based rehabilitation, and simulates real-world practice to demonstrate the clinical relevance of acupuncture as an adjunctive treatment. Consequently, effect sizes should be interpreted as the added value of acupuncture to a standard cognitive program, with the differences controlled via sham stimulation directly attributable to the specific neural stimulation mechanisms of acupoints.

**Limitations and Future Directions**

While the randomized controlled trial and blinding methodologies are upheld, the feasibility of achieving total blindness has been acknowledged as challenging, particularly in nondrug trials. Given the unique nature of acupuncture treatment, a double-blind procedure is not feasible, leading to the decision to blind outcome evaluators and statistical analysts. In addition, both groups receive conventional cognitive training to ensure ethical equivalence; however, this may reduce the contrast between groups. Moreover, we acknowledge the limitations in generalizability of our study findings, primarily due to the single-center design and the constraints imposed by resource-intensive research protocols and the standardization of millimeter-level interventions. To minimize potential biases, we have implemented rigorous quality control measures. For future research, we plan to conduct multicenter trials to quantitatively assess general applicability and conduct additional real-world validation to further enhance the generalizability of our findings.

In summary, by adopting a multimodal neuroimaging approach, this study delves into the impact of acupuncture on the brain structure and function of patients with PSCI. By examining correlations with changes in cognitive function, this study aims to clarify the specific targets and mechanisms through which acupuncture enhances cognitive function. The findings are anticipated to yield reliable imaging markers for evaluating the efficacy of acupuncture and prognosis of PSCI, offering valuable clinical applications. If this study demonstrates a significant intervention effect, it will contribute robust evidence supporting acupuncture’s ability to ameliorate cognitive dysfunction in patients with PSCI, providing insights into its neuroimaging mechanisms.

## Abbreviations

DMN: default mode network
DSMB: data safety monitoring board
DTI: diffusion tensor imaging
fMRI: functional magnetic resonance imaging
FOV: field of view
MCI: mild cognitive impairment
MD: mean difference
MMSE: Mini-Mental State Examination
MoCA: Montreal Cognitive Assessment
MRI: magnetic resonance imaging
PSCI: poststroke cognitive impairment
ROI: region of interest
TCM: traditional Chinese medicine
TE: echo time
TR: repetition time
